# Cardiopulmonary nematodes of wild carnivores from Denmark: Do they serve as reservoir hosts for infections in domestic animals?

**DOI:** 10.1016/j.ijppaw.2020.08.001

**Published:** 2020-08-15

**Authors:** Louise Lemming, Ann Cholewa Jørgensen, Linette Buxbom Nielsen, Stine Thorsø Nielsen, Helena Mejer, Mariann Chriél, Heidi Huus Petersen

**Affiliations:** a Centre for Diagnostic, Technical University of Denmark, Kemitorvet, DK-2800, Kgs. Lyngby, Denmark; b Parasitology and Aquatic Pathobiology, Department of Veterinary and Animal Sciences, University of Copenhagen, Dyrlægevej 100, DK-1870, Frederiksberg C, Denmark

**Keywords:** Cardiopulmonary nematodes, Necropsy, Molecular analysis, Red foxes, Raccoon dogs, American mink, Eurasian otters, Beech martens

## Abstract

The cardiopulmonary nematodes *Angiostrongylus vasorum, Crenosoma vulpis, Capillaria aerophila* and *Aelurostrongylus abstrusus*, are a cause of concern in the scientific and veterinary community, potentially causing significant disease in domestic animals. To investigate the potential of wild carnivores as reservoir hosts to these parasites, a total of 1041 animals from seven regions of Denmark were sampled: 476 raccoon dogs (*Nyctereutes procyonoides*), 367 red foxes (*Vulpes vulpes*), 123 American mink (*Neovison vison*), 31 beech martens (*Martes foina*), 30 Eurasian otters (*Lutra lutra*) and 14 polecats (*Mustela putorius*). Hearts and lungs were collected and examined for cardiopulmonary parasites. *Capillaria aerophila* was identified using morphology, whereas *A. vasorum* and *C. vulpis* were identified by a duplex real-time PCR, and *A. abstrusus* by conventional PCR. This is the first Danish report of *A. vasorum* and *C. vulpis* infections in raccoon dogs, mink and polecats, and of *C. aerophila* in raccoon dogs and beech martens. In addition, this is the first time *A. vasorum* and *C. vulpis* have been identified in wild animals from the island of Bornholm, just as it is the first report of *C. vulpis* in American mink, and *C. vulpis* and *A. vasorum* in polecats in Europe. The prevalence of *A. vasorum* appears to have increased in red foxes in Denmark compared to previous studies, while *C. vulpis* and *C. aerophila* prevalences are lower. Our data show that several wild carnivores can serve as reservoir hosts for *A. vasorum, C. vulpis* and *C. aerophila* in Denmark, and that *A. vasorum* appears more abundant than previously reported. It is speculated that the *A. vasorum* increase might relate to increased snail abundance, which may be due to a rise in mean yearly temperatures in Denmark.

## Highlights

• The first report of *C. vulpis* in American mink.
• The first report of *C. vulpis* and *A. vasorum* in polecats.
• The first report of *A. vasorum* in raccoon dogs, mink, and polecats from Denmark.
• The first report of *C. vulpis* in raccoon dogs, mink, and polecats from Denmark.
• The first report of *C. aerophila* in raccoon dogs and beech martens from Denmark.

## Graphical abstract

**Figure:**
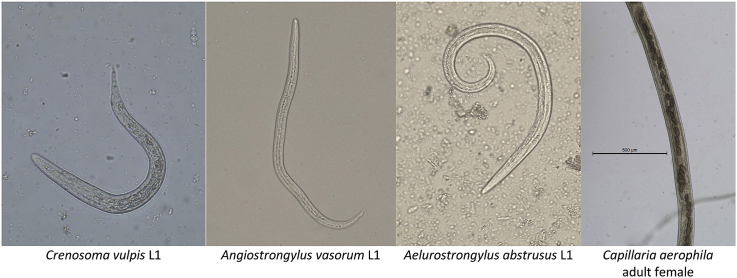

## 1 Introduction

The cardiopulmonary nematodes *Angiostrongylus vasorum*, *Crenosoma vulpis*, *Capillaria aerophila* (synonym *Eucoleus aerophilus*) and *Aelurostrongylus abstrusus*, have gained increased attention since recent introduction into previously non-endemic areas (Demiaszkiewicz et al., 2014; Hurníková et al., 2013; Jolly et al., 2015; Simin et al., 2014). Canine angiostrongylosis can be severe and even fatal (Conboy, 2009; Koch and Willesen, 2009), whereas *C. vulpis* and *C. aerophila* are less pathogenic, typically characterized by chronic cough (Burgess et al., 2008; Conboy, 2009; Reilly et al., 2000; Rinaldi et al., 2007), therefore updated knowledge of parasite distribution is of particular importance to veterinary clinicians.

*Angiostrongylus vasorum* and *C. vulpis* commonly infect red foxes (*Vulpes vulpes*) and domestic dogs (*Canis familiaris*), though infection of several wild canids and mustelids are possible (Anderson, 2000; Traversa et al., 2010). *Capillaria aerophila* are identified from red foxes, wild mustelids, domestic cats (*Felis catus*) and dogs (Otranto et al., 2015; Traversa and Cesare, 2014), and occasionally humans (Lalošević et al., 2013). *Aelurostrongylus abstrusus* commonly infect domestic cats (Giannelli et al., 2017), and feral felids (Conboy, 2009; Elsheikha et al., 2016; Penagos-Tabares et al., 2018). In Spain, *Aelurostrongylus* spp. has been documented in American mink (*Neovison vison*) (Martínez-Rondán et al., 2017), suggesting that mink can act as reservoir hosts. With few exceptions, in Denmark *A. vasorum, C. vulpis* and *C. aerophila* are only documented from red foxes (Koch and Willesen, 2009; Saeed et al., 2006; Willingham et al., 1996); a badger (*Meles meles*) infected with *C. aerophila* (Dietz et al., 1998), an Eurasian otter (*Lutra lutra*) infected with *A. vasorum* (Madsen et al., 1999), and feral cats infected with *C. aerophila* (Olsen et al., 2015). *Aelurostrongylus abstrusus* infections have been reported in domestic and feral cats from Denmark (Olsen et al., 2015).

Previous Danish studies on cardiopulmonary nematodes in wildlife are based solely on morphological identification (Al-Sabi et al., 2014; Saeed et al., 2006; Willingham et al., 1996). However, accurate morphological identification can be problematic due to fragmented nematodes, poor organ quality as animals have been shot in the thoracic region, confounding debris obscuring larvae and partial decomposition of the carcasses and thus the parasites. Moreover, the previous Danish publications includes only a single wildlife species. Therefore, the current role of wildlife as reservoir hosts for cardiopulmonary nematodes in Denmark is unknown. In addition, several Danish veterinary clinics report increasing incidence of canine angiostrongylosis outside the metropolitan area of Copenhagen and North Zealand (personal communication). North Zealand, including Copenhagen, has been considered a hyperendemic focus of *A. vasorum* in red foxes and domestic dogs for decades (Bolt et al., 1992; Saeed et al., 2006). The increase in canine angiostrongylosis outside the hyperendemic area might relate to an increased prevalence in wildlife over the past decade as observed for *A. vasorum* in Great Britain (Taylor et al., 2015) and Switzerland (Gillis-Germitsch et al., 2020).

Red foxes, raccoon dogs (*Nyctereutes procyonoides*) and American mink (hereafter named mink, as European mink (*Mustela lutreola*) have never been recorded in Denmark) are abundant wildlife species in Denmark and could contribute to substantial contamination with environmental stages of cardiopulmonary nematodes. Red foxes are native to Denmark and the most abundant species distributed throughout the country (apart from the island of Bornholm and some smaller islands) with a population size of approx. 40,000–60,000 individuals (Asferg et al., 2013). The raccoon dog is an invasive species in Denmark. It was first recorded in 1980, and the population has increased significantly since then to approx. 2000–3000 individuals (The Environmental Protection Agency, n.d.). Mink were originally imported to Denmark for fur production and kept in captivity. Today, mink can be found in the wild all over Denmark due to escapees from farms (Pagh et al., 2019), but the population size is unknown. Eurasian otters, beech martens (*Martes foina*) and polecats (*Mustela putorius*) are believed to be less common.

The aim of this study was to investigate the potential of six Danish wild carnivore species as reservoir hosts for *A. vasorum, C. vulpis, C. aerophila*, and *A. abstrusus* in seven different Danish regions.

## 2 Materials and methods

### 2.1 Study animals

Carcasses from 1041 wild carnivores; raccoon dogs (n = 476), red foxes (n = 367), mink (n = 123), beech martens (n = 31), Eurasian otters (n = 30) and polecats (n = 14) were examined for cardiopulmonary nematodes (Table 1).

**Table 1:** Number of animals and organs examined, and number and total percentage of animals positive for cardiopulmonary nematodes (with 95% confidence intervals) per animal species.

Animal species	Animals	Lungs	Hearts	Positive	% positive
	examined			animals	[95% CI]
Raccoon dog (*Nyctereutes procyonoides*)	476	473	467	48	10.1
					[7.6–13.2]
Red fox (*Vulpes vulpes*)	367	363	357	82	22.3
					[18.2–27.0]
American mink (*Neovison vison*)	123	122	121	18	14.6
					[9.1–22.4]
Beech martens (*Martes foina*)	31	30	28	6	19.4
					[8.1–38.1]
Eurasian otters (*Lutra lutra*)	30	29	28	1	3.3
					[0.2–19.1]
Polecats (*Mustela putorius*)	14	14	13	14	100.0
					[73.2–100.0]
**Total**	**1041**	**1031**	**1014**	**169**	**16.2**
					**[14.1**–**18.6]**

**Fig. 1:**
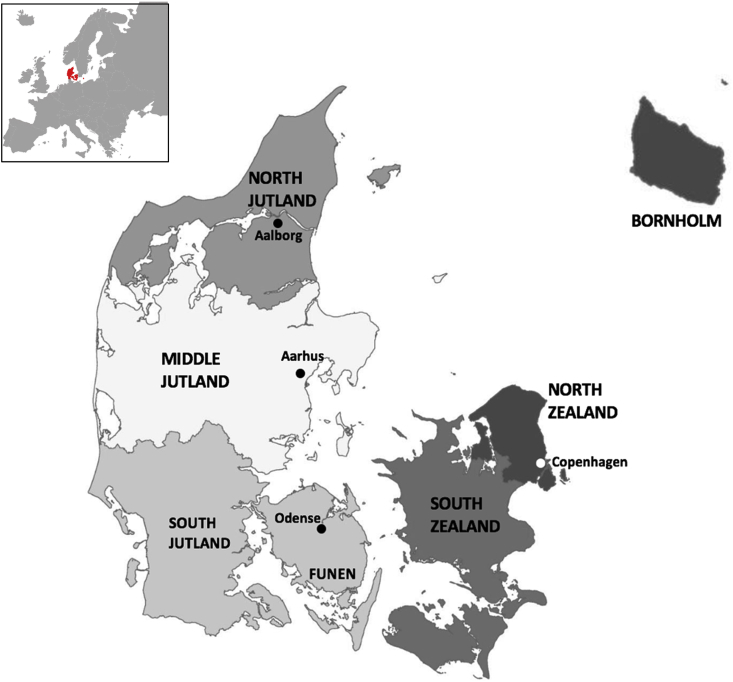
Map of Denmark showing Denmark's position in Europe and a map of Denmark showing the regional division and the four major cities.

The carcasses were submitted for necropsy between January 2017 and March 2018, as a part of the national wildlife surveillance program. The carnivores were hunted, found dead or euthanized for animal welfare reasons. All animals were transported in sealed plastic bags and subsequently stored at −80 °C for min. 4 days prior to necropsy to inactivate potential zoonotic parasites. The wild carnivores were divided into seven regional groups depending on their origin: North Jutland (NJ), Middle Jutland (MJ), South Jutland (SJ), Funen (FU), North Zealand including Copenhagen (NZ), South Zealand (SZ) and Bornholm (BO) (Fig. 1). Funen, Zealand and Bornholm are all major populated islands, while Jutland is a peninsula extending from north Germany.

At necropsy, hearts and lungs were collected and stored at −20 °C until further examination. Occasionally, hearts or lungs were unsuitable for examination due to traumatic injury, predation or decomposition. Individual data on regional origin, and gender were recorded when possible.

### 2.2 Examination of hearts and lungs

The heart was separated from the lungs by transecting all the arteries and veins proximal to the base of the heart. The ventricles and major blood vessels were opened using scissors and the heart was examined microscopically for adult worms using a stereomicroscope (Leica microsystems GmBH, Germany) at 80–100× magnification. The pulmonary blood vessels, trachea, and bronchial tree were dissected using scissors and inspected macroscopically for adult worms. Parallel incisions were made throughout the lung lobes approximately 1–2 cm apart. Subsequently, the heart and lungs were flushed with 500 mL tap water into a 500 mL conical glass. The fluid was left to sediment for 20 min. After which, the supernatant was removed, and the sediment transferred to microscopic slides, for examination using a light microscope (Leica DM2500 LED optical microsystems GmBH, Germany) at 100–400× magnification. All worms and larvae, except adult worms of *C. aerophila*, from individual animals were collected in 2 mL eppendorf tubes and stored at −20 °C. Adult worms of *C. aerophila* were identified morphologically based on their easily identifiable characteristics, as described by Christenson (1935). Samples co-infected with *C. aerophila* and other cardiopulmonary parasites were subjected to DNA extraction and molecular analysis.

### 2.3 DNA extraction of parasites

From each positive animal, up to 30 larvae or two adult worms or worm fragments were distributed into 1.5 mL eppendorf tubes, i.e. some samples were distributed into >1 tube and all tubes were analysed for each animal. From a pilot study, it was verified that >30 larvae or >2 adult worms could saturate the DNA extraction columns. Genomic DNA was extracted from all eppendorf tubes using the QIAmp DNA Mini Kit (Qiagen, Hilden, Germany) according to the manufacturer's instructions. Extracted DNA was eluted in a final volume of 100 μL AE Buffer (QIAmp DNA Mini Kit, Qiagen, Hilden, Germany) and stored at −20 °C.

### 2.4 Molecular analysis

#### 2.4.1 Molecular identification of *Angiostrongylus vasorum* and *Crenosoma vulpis*

*Angiostrongylus vasorum* and *C. vulpis* were identified using a modified method by Schug et al. (2018). In brief, duplex real-time PCT (rt-PCR) was used to target the ribosomal DNA (rDNA) of the internal transcribed spacer 2 (ITS-2) region, with primer and probe sequences listed in Table 2. PCR amplification was carried out in a total volume of 20 μl, containing 0.5 μM of each primer, 0.2 μM of each probe, 10 μL JumpStart™ Taq ReadyMix™ for Quantitative PCR (Sigma-Aldrich), 2.5 mM MgCl_2_, 2.0 μL DNA, and dH_2_O. PCR reactions were performed in a Rotor-Gene Q rt-PCR cycler (Qiagen, Hilden, Germany) using the following conditions: 94 °C for 2 min, followed by 45 cycles of 94 °C for 15 s, 55 °C for 60 s, 1 min with fluorescence detection in the green (FAM) and yellow (HEX) channel. DNA from 15 *A. vasorum* first stage larvae (L1) obtained from a German domestic dog, and DNA from 15 *C. vulpis* L1 obtained from a Swedish domestic dog were used as positive controls.

**Table 2:** Primer and probe sequences used in PCR assays.

Parasite	Sequence	Partial gene	Base pair length	Reference
***A. vasorum***			121	a
**Primer**				
I2F10 (forward)	5′-CGCATGATGAAAGAATGCTG-3′	ITS-2		
I2R9 (reverse)	5′-GACGACGACGACAACCACT-3′	ITS-2		
**Probe**				
I2P2	FAM-ACAACATTGCTTGTCGAACGGCGTT-BHQ1			
***C. vulpis***			~90	a
**Primer**				
CvITS2f (forward)	5′-GCATGATATTCGACGATTG-3′	ITS-2		
CvITS2r (reverse)	5′-GTGTGATCTAGTCATGTATAAC-3′	ITS-2		
**Probe**				
CvITS2p	HEX-CAGCAATGAGAAGACACTATACACAAG-BHQ1			
***A. abstrusus***			~233	b
AabFor (forward)	5′-GTAACAACGATATTGGTACTATG-3′	ITS-2		
AabRev (reverse)	5′-GAACTCCTTCACGTGCTACTCG-3′	ITS-2		

a Schug et al. (2018).

b Traversa and Guglielmini (2008).

#### 2.4.2 Molecular identification of *Aelurostrongylus abstrusus*

Examination for *A. abstrusus* was performed by conventional PCR according to Traversa and Guglielmini (2008) with minor modifications. The ITS-2 region of ribosomal DNA (~233 base pairs) was amplified using the primers listed in Table 2. PCR amplifications were carried out in a final volume of 50 μL containing: 100 pmol of each primer, 25.0 μL RED*Taq* ReadyMix (Sigma-Aldrich, Chemie, GmbH), 5.0 μl template DNA, and dH_2_O. Amplifications were performed using a Biometra T3 Thermocycler using the following conditions: 94 °C for 7 min followed by 40 cycles of 94 °C for 45 s, 50 °C for 45 s, and 72 °C for 45 s, and a final extension for 10 min at 72 °C. DNA from 15 *A. abstrusus* L1 obtained from a Danish domestic cat was used as a positive control.

Amplicons were electrophoresed in a 2% agarose E-Gel™ (Invitrogen, Thermo Fisher Scientific, Denmark) and lengths were sized by comparison with a 100-bp DNA Ladder (New England BioLabsInc., Ipswich, England).

#### 2.4.3 Efficacy of PCR assays

For each parasite species, the detection limits were established using a duplex dilution series of MQ water spiked with 1, 2, 5, 10, 15, and 30 L1 isolated from the same material that was used for positive controls (see section 2.4.1 and 2.4.2). The rt-PCR consistently detected *A. vasorum* and *C. vulpis* DNA in samples containing ≥2 and ≥5 L1, respectively. The conventional PCR consistently detected *A. abstrusus* in samples containing ≥1 L1.

### 2.5 Data analysis

The prevalence of cardiopulmonary parasites and 95% confidence intervals were calculated for each carnivore species and separately for their region of origin.

## 3 Results

Table 1 gives an overview of the wild carnivores included in the study, the number of examined organs and animals positive for cardiopulmonary nematodes. Adult *C. aerophila* were morphologically identified in 50 carnivores (4.8%), of which 19 were co-infected with other cardiopulmonary parasites. These were subjected to DNA extraction together with other parasites from a further 119 carnivores (n = 138). In total, DNA was successfully extracted from 119/138 samples and determined to belong to *A. vasorum* and *C. vulpis*. The remaining 19 samples have been designated as positive for unidentified cardiopulmonary parasites. The prevalences and 95% CI of *A. vasorum*, *C. vulpis, C. aerophila* and unidentified nematodes by carnivore species and region of origin are listed in Table 3.

**Table 3:** Parasite prevalence (with 95% confidence interval) based on biomolecular identification according to host species and region. NJ = North Jutland, MJ = Middle Jutland, SJ = South Jutland, FU = Funen, NZ = North Zealand, SZ = South Zealand and BO = Bornholm, U = unknown region, DK = Denmark, total countrywide prevalence.

*A. vasorum*	Host species																																																			
Region	Red fox						Raccoon dog							Mink									Beech Marten								Polecat								Eurasian otter							Total						
	No. pos		*% pos*				No. pos			% pos				No. pos							% pos		No. pos				% pos				No. pos				% pos				No. pos			% pos				No. pos						% pos
	/total		*[95% CI]*				/total			[95% CI]				/total							[95% CI]		/total				[95% CI]				/total				[95% CI]				/total			[95% CI]				/total						[95% CI]
*NJ*	2/88		2.3				1/47			2.1				0/10							–		0/8				–				1/1				10.0				0/8			–				4/162						2.5
			[0.4–8.7]							[0.1–12.7]																									[5.5–100]																	[0.8–6.6]
*MJ*	3/161		1.9				7/195			3.6				0/9							–		0/16				–				2/4				5.0				0/9			–				11/394						2.8
			[0.5–5.8]							[1.6–7.6]																									[9.2–90.8]																	[1.5–5.1]
*SJ*	1/59		1.7				7/234			3.0				0/1							–		0/4				–				–				–				0/10			–				7/308						2.3
			[0.09–10.3]							[1.3–6.3]																																										[1.0–4.8]
*FU*	1/4		25.0				–			–				0/11							–		–				–				–				–				0/1			–				1/15						6.7
			[1.3–78.1]																																																	[0.4–34.0]
*NZ*	6/16		37.5				–			–				0/2							–		–				–				–				–				–			–				6/18						33.3
			[16.3–64.1]																																																	[14.4–58.8]
*SZ*	10/27		37.0				–			–				0/1							–		0/2				–				4/9				44.4				–			–				14/39						35.9
			[20.1–57.5]																																[15.3–77.3]																	[21.7–52.9]
*BO*	–		–				–			–				1/86							1.2		–				–				–				–				–			–				1/86						1.2
																					[0.1–7.2]																															[0.06–7.2]
*U*	2/12		16.7				–			–				0/3							–		0/1				–				–				–				0/2			–				2/18						11.1
			[2.9–49.1]																																																	[2.0–36.1]
***Total***	**25/367**		**6.8**				**15/476**			**3.2**				**1/123**							**0.8**		**0/31**				**-**				**7/14**				**5.0**				**0/30**			**-**				**48/1041**						**4.6**
			**[4.5**–**10.0]**							**[1.8**–**5.3]**											**[0.0**–**5.1]**														**[24.0**–**76.0]**																	**[3.5**–**6.1]**
***C. vulpis***^a^	**Host species**																																																			
***Region***	***Red fox***					***Raccoon dog***							***Mink***											***Beech Marten***								***Polecat***									***Eurasian otter***								***Total***			
	***No. pos***	***% pos***				***No. pos***			***% pos***				***No. pos***						***% pos***					***No. pos***				***% pos***				***No.pos***					***% pos***				***No. pos***			***% pos***			***No. pos***					***% pos***
	***/total***	***[95% CI]***				***/total***			***[95% CI]***				***/total***						***[95% CI]***					***/total***				***[95% CI]***				***/total***					***[95% CI]***				***/total***			***[95% CI]***			***/total***					***[95% CI]***
*NJ*	2/87	2.3				0/47			–				0/10						–					0/8				–				0/1					–				0/7			–			2/160					1.3
		[0.4–8.8]																																																		[0.2–4.9]
*MJ*	12/158	7.6				7/194			3.6				0/9						–					0/15				–				0/4					–				0/9			–			19/389					4.9
		[4.2–13.2]							[1.6–7.6]																																											[3.0–7.7]
*SJ*	1/59	1.7				18/232			7.8				0/1						–					0/4				–				–					–				0/10			–			19/306					6.2
		[0.1–10.3]							[4.8–12.2]																																											[3.9–9.7]
*FU*	0/4	–				–			–				0/11						–					–				–				–					–				0/1			–			0/16					
*NZ*	3/16	18.8				–			–				0/2						–					–				–				–					–				–			–			3/18					1.7
		[5.0–46.3]																																																		[4.4–42.3]
*SZ*	1/27	3.7				–			–				0/1						–					0/2				–				1/9					11.1				–			–			2/39					5.1
		[0.1–20.9]																																			[0.6–49.3]															[0.9–18.3]
*BO*	–	–				–			–				7/85						8.2					–				–				–					–				–			–			7/85					8.3
																			[3.7–16.8]																																	[3.7–16.8]
*U*	1/12	8.3				–			–				0/3						–					0/1				–				–					–				0/2			–			1/18					5.6
		[0.4–40.2]																																																		[0.3–29.4]
***Total***	**20/363**	**5.5**				**25/473**			**5.3**				**7/122**						**5.7**					**0/30**				–				**1/14**					**7.1**				**0/29**			–			**53/1031**					**5.1**
***DK***		**[3.5**–**8.5]**							**[3.5**–**7.8]**										**[2.5**–**11.9]**																		**[0.4**–**35.8]**															**[3.9**–**6.7]**
***C. aerophila***^a^	**Host species**																																																			
***Region***	***Red fox***							***Raccoon dog***							***Mink***										***Beech Marten***								***Polecat***							***Eurasian otter***								***Total***				
	***No. pos***			***% pos***				***No. pos***			***% pos***				***No. pos***					***% pos***					***No. pos***				***% pos***				***No. pos***			***% pos***				***No. pos***			***% pos***					***No. pos***			***% pos***	
	***/total***			***[95% CI]***				***/total***			***[95% CI]***				***/total***					***[95% CI]***					***/total***				***[95% CI]***				***/total***			***[95% CI]***				***/total***			***[95% CI]***					***/total***			***[95% CI]***	
*NJ*	12/87			13.8				1/47			2.1				0/10					–					0/8				–				0/1			–				0/7			–					13/160			8.1	
				[7.6–23.2]							[0.1–12.7]																																								[4.6–13.8]	
*MJ*	17/158			10.8				3/194			1.6				0/9					–					0/15				–				0/4			–				0/9			–					20/389			5.1	
				[6.7–16.9]							[0.4–4.8]																																								[3.3–8.0]	
*SJ*	7/59			11.9				5/232			2.2				0/1					–					0/4				–				–			–				0/10			–					12/306			3.9	
				[5.3–23.5]							[0.8–5.2]																																								[2.1–6.9]	
*FU*	0/4			–				–			–				0/11					–					–				–				–			–				0/1			–					0/16			–	
*NZ*	2/16			12.5				–			–				0/2					–					–				–				–			–				–			–					2/18			11.1	
				[2.2–39.6]																																															[2.0–36.1]	
*SZ*	1/27			3.7				–			–				0/1					–					2/2				100				0/9			–				–			–					3/39			7.7	
				[0.2–20.9]																									[19.8–100]																						[2.0–22.0]	
*BO*	–			–				–			–				0/85					–					–				–				–			–				–			–					0/85			–	
*U*	0/12			–				–			–				0/3					–					0/1				–				–			–				0/2			–					0/18			–	
***Total***	**39/363**			**10.7**				**9/473**			**1.9**				**0/122**										**2/30**				**6.7**				**0/14**			–				**0/29**			**-**					**50/1031**			**4.8**	
***DK***				**[7.8**–**14.5]**							**[0.9**–**3.7]**																		**[1.2**–**23.5]**																						**[3.7**–**6.4]**	
*Unidenfied lungworms*	***Red fox***							***Racoon dog***								***Mink***									***Beech Marten***								***Polecat***							***Eurasian otter***								***Total***				
	***No. pos***				***% pos***			***No. pos***				***% pos***					***No. pos***					***% pos***				***No. pos***				***% pos***				***No. pos***				***% pos***		***No. pos***					***% pos***			***No. pos***		***% pos***		
	***/total***				***[95% CI]***			***/total***				***[95% CI]***						***/total***				***[95% CI]***				***/total***				***[95% CI]***				***/total***				***[95% CI]***		***/total***					***[95% CI]***			***/total***		***[95% CI]***		
Total	9/367				2.5			4/476				0.8						0/123				–				4/31				12.9				2/14				14.3		0/30					–			19/1041		1.8		
					[1.3–4.6]							[0.3–2.1]																		[5.1–28.9]								[4.0–40.0]												[1.2–2.8]		

a *In 10 animals, lungs were missing and the heart was the only organ examined. These animals were only examined for A. vasorum*.

*Angiostrongylus vasorum* and *C. vulpis* were identified from red foxes, raccoon dogs, mink and polecats, while *C. aerophila* was identified from red foxes, raccoon dogs and beech martens. Otters were negative for all parasite species. None of the carnivores were positive for *A. abstrusus*.

Co-infections with *A. vasorum* and *C. vulpis* were observed in 11 carnivores (six raccoon dogs and five red foxes), four were co-infected with *A. vasorum* and *C. aerophila* (one raccoon dog and three red foxes) and three were co-infected with *C. vulpis* and *C. aerophila* (three red foxes).

## 4 Discussion

This study confirmed the occurrence of *A. vasorum, C. vulpis* and *C. aerophila* in red foxes, but also provided novel evidence of these parasites from other wildlife species, confirming their potential as reservoir host for domestic animals in Denmark. Thus, we report, for the first time, infection with *A. vasorum* and *C. vulpis* in raccoon dogs, mink and polecats, and *C. aerophila* infection in raccoon dogs and beech martens from Denmark. Additionally, this is the first report of *A. vasorum* and *C. vulpis* in wild carnivores from the island of Bornholm. Lastly, this is, to our knowledge, the first report of *C. vulpis* infection in mink, and *C. vulpis* and *A. vasorum* infections in polecats worldwide. *Aelurostrongylus abstrusus* was absent from all examined animals.

*Angiostrongylus vasorum* and *C. vulpis* prevalences in raccoon dogs from Denmark (2.1–3.6% and 3.6–7.8%, respectively) were similar to those observed for red foxes from the same regions (1.7–2.3% and 1.7–7.6%, respectively). Similarity in the regional prevalence of *A. vasorum* and *C. vulpis* was observed for both red foxes and raccoon dogs. Both parasites have previously been identified in raccoon dogs in other European countries (Bružinskaitė-Schmidhalter et al., 2012; Laurimaa et al., 2016; Shimalov and Shimalov, 2002; Thiess et al., 2001), and have probably been present in raccoon dogs since they first came to Denmark in 1980. Raccoon dogs are currently only present in Jutland (the peninsula of Denmark) and with a few individuals on the island of Funen. However, the population continuous to increase despite a large effort from local hunters and the authorities to control population numbers. Based on the prevalence and the increasing population size, raccoon dogs might constitute an important reservoir for *A. vasorum* and *C. vulpis* infections in domestic animals in Jutland. Fortunately, this may be counteracted by the raccoon dog's behaviour as they tend to defecate in “latrines”, hibernate during winter and avoid human contact (Al-sabi et al., 2009). This may diminish scattered environmental contamination with the parasites near domestic animals, but does not reduce exposure for other wildlife species that may bring the infections closer to domestic animals.

*Angiostrongylus vasorum* and *C. vulpis* were recorded in red foxes with regional prevalences of 1.7–37.5% and 1.7–18.8%, respectively (Table 3), demonstrating that these parasites are common in red foxes from Denmark. The red fox abundance is significantly higher than raccoon dogs and polecats, although the true population sizes are unknown. Moreover, red foxes are distributed throughout most of the country, both in rural and urban areas, and are active year-round (Al-sabi et al., 2009). In urban areas, red foxes often defecate in private gardens and city parks, areas where intermediate hosts are common and domestic dogs are walked. Due to behavioural habits, abundance and parasite prevalences, red foxes are most likely the main reservoir host for cardiopulmonary infections in domestic dogs in Denmark.

Polecats also constitute a previously unknown reservoir for *A. vasorum* and *C. vulpis* in Denmark with prevalences of 5.0% and 7.1%, respectively. *Angiostrongylus vasorum* has previously been identified from other small mustelids (Simpson et al., 2016), but neither *A. vasorum* nor *C. vulpis* infections in polecats have previously been documented. As only 14 polecats were included in this study, further investigations with larger sample size is needed to investigate the importance of this species as a reservoir host.

We observed *C. aerophila* in raccoon dogs and beech martens from Denmark for the first time, although with relatively low prevalences in raccoon dogs. *Capillaria aerophila* was more prevalent in red foxes (10.7%) compared to beech martens (6.7%) and raccoon dogs (1.9%) suggesting that red foxes are the most important reservoir host. In contrast to *A. vasorum* and *C. vulpis*, *C. aerophila* prevalences in red foxes were similar (10.8%–13.8%) in four out of five regions. These findings concur with previous reported prevalences for red foxes in South Jutland and North Zealand (Al-Sabi et al., 2014). Evenly distributed *C. aerophila* prevalences in different geographical areas have also been reported from other countries (Davidson et al., 2006; Hodžíc et al., 2016; Morgan et al., 2008; Schug et al., 2018). However, prevalences reported in former Danish studies were considerably higher (87.1% and 93.8%, respectively) (Al-Sabi et al., 2014) than in our study, which may reflect differences in sampling procedures (see below). However, fluctuation in *C. aerophila* prevalences from year to year has been observed in red foxes from Switzerland (Gillis-Germitsch et al., 2020), while it has remained constant in other countries (Morgan et al., 2008; Sréter et al., 2003; Taylor et al., 2015; Tolnai et al., 2015).

The *A. vasorum* prevalence in red foxes was, as expected, considerably higher (37.5%) in North Zealand compared to red foxes from Jutland (1.7–2.3%), which is in line with previous reports in this area (35.9–80.0%) (Al-Sabi et al., 2014; Bolt et al., 1992; Saeed et al., 2006; Willingham et al., 1996). North Zealand has been considered an *A. vasorum* hyperendemic area for decades (Bolt et al., 1992; Saeed et al., 2006). Originally, *A. vasorum* was introduced to North Zealand with a domestic dog, which was infected in France (Finnerup, 1983). Thereafter, the parasite seemingly spread to other domestic dogs and red foxes in the area. Unexpectedly, *A. vasorum* prevalence in red foxes originating from South Zealand was equal to the prevalence in the hyperendemic North Zealand. This new finding indicates that the hyperendemic area has expanded to include all of Zealand. In contrast, *A. vasorum* prevalence in red foxes from Jutland was significantly lower. This geographical difference between Jutland and Zealand could include variation in intermediate host density and density of the final host. Nevertheless, the prevalence in red foxes originating from Jutland was slightly higher, and prevalence in red foxes originating from South Zealand substantially higher than previously reported for these areas (Al-Sabi et al., 2014; Bolt et al., 1992; Jacobsen, 2007; Saeed et al., 2006; Willingham et al., 1996). Our results are in line with observations from other European countries: For example, *A. vasorum* prevalence in red foxes in Switzerland has increased 4-fold in 5 years (Gillis-Germitsch et al., 2020), in Hungary, 3.6-fold in 12 years (Sréter et al., 2003; Tolnai et al., 2015), and 2.6-fold in 8 years in Great Britain (Taylor et al., 2015). This increase over time might relate to increased snail abundance. Snail abundance is temperature dependent, e.g. snail egg development requires at least 10 °C for *Arion lusitanicus* (Kozłowski, 2007), a common intermediate host for *A. vasorum* (Ferdushy et al., 2009). Moreover, parasite larvae development in the intermediate host is also dependent on temperature which can impact transmission of the parasite (Ferdushy et al., 2010). In Denmark, the mean yearly temperature has increased the past years (Danmarks statistik, 2014). Moreover, foxes do not develop protective immunity against *A. vasorum* and can be re-infected (Woolsey et al., 2017).

As for *A. vasorum*, *C. vulpis* was more prevalent in red foxes from North Zealand (18.8%) compared to those from Jutland (1.7–7.6%). This contrasts findings from a previous Danish study where *C. vulpis* prevalence in red foxes from South Jutland and Copenhagen metropolitan area (in our study, animals from the Copenhagen metropolitan area is included in the area designated North Zealand) was equal (22.9%) (Al-Sabi et al., 2014). Thus, *C. vulpis* prevalence in our study is low in South Jutland compared to previous Danish study by Al-Sabi et al. (2014). Likewise, *C. vulpis* prevalence in red foxes in our study is lower than previously reported in Europe (23.3%–53.8%) (Bružinskaitė-Schmidhalter et al., 2012; Schug et al., 2018). Besides variations in final- and intermediate host abundance, the variation could relate to differences in diagnostic methods (Hodžíc et al., 2016; Morgan et al., 2008). The method used herein for parasite collection has some limitations. Only a moderate amount of water was used to flush the organs compared to other studies, lung lobes were examined with parallel incisions, the trachea and bronchi were not all scraped, and blood clots were not dissolved in water as has previously been reported (Davidson et al., 2006; Hodžíc et al., 2016; Schug et al., 2018). All of which may have reduced the sensitivity of the method leading to the lower prevalences observed for *C. vulpis* and *C. aerophila* compared to previous studies.

*Aelurostrongylus abstrusus* was absent from our study sample population, suggesting that wild carnivores can be considered unimportant as reservoir hosts for this parasite. This confirms that the parasite is rather species-specific (Otranto et al., 2015; Traversa et al., 2010), even though there is a recent report on *Aelurostrongylus* spp. in mink (Martínez-Rondán et al., 2017).

Mink is the only carnivore present on the island of Bornholm. In total, 8.2% of these was infected with *C. vulpis* and 1.2% with *A. vasorum*. Interestingly, mink from other regions were not infected with these parasites. No mink were infected with *C. aerophila*. As Bornholm is an island, parasites were likely introduced through importation of infected animals, notably domestic dogs. As prevalences were low in mink, their role as reservoir hosts can be considered negligible.

## 5 Conclusion

Our findings showed for the first time that raccoon dogs, mink, and polecats from Denmark can be infected with *A. vasorum* and *C. vulpis,* and that raccoon dogs and beech martens were infected with *C. aerophila.* Our findings confirm that other wild carnivores in addition to red foxes can act as reservoirs for cardiopulmonary nematodes in domestic animals. Furthermore, prevalences of *A. vasorum* have increased in red foxes in most areas compared to previous studies, while quantitatively and epidemiologically, the role of these reservoir hosts is unknown.

## Declaration of competing interest

None.
